# Tuberculosis among Health Care Workers

**DOI:** 10.3201/eid1703.100947

**Published:** 2011-03

**Authors:** Iacopo Baussano, Paul Nunn, Brian Williams, Emanuele Pivetta, Massimiliano Bugiani, Fabio Scano

**Affiliations:** Author affiliations: University “A. Avogadro,” Novara, Italy (I. Baussano);; Centro per la Prevenzione Oncologica–Piemonte, Novara (I. Baussano);; Imperial College, London, UK (I. Baussano);; World Health Organization, Geneva, Switzerland (P. Nunn, B. Williams, F. Scano);; University of Turin, Turin, Italy (E. Pivetta);; Centro per la Prevenzione Oncologica–Piemonte, Turin (E. Pivetta);; Regional Reference Centre for Tuberculosis Prevention, Turin (M. Bugiani)

**Keywords:** Tuberculosis and other mycobacteria, health care workers, systematic review, research

## MEDSCAPE CME

Medscape, LLC is pleased to provide online continuing medical education (CME) for this journal article, allowing clinicians the opportunity to earn CME credit.

This activity has been planned and implemented in accordance with the Essential Areas and policies of the Accreditation Council for Continuing Medical Education through the joint sponsorship of Medscape, LLC and Emerging Infectious Diseases. Medscape, LLC is accredited by the ACCME to provide continuing medical education for physicians.

Medscape, LLC designates this Journal-based CME activity for a maximum of 1 *AMA PRA Category 1 Credit(s)*^TM^. Physicians should claim only the credit commensurate with the extent of their participation in the activity.

All other clinicians completing this activity will be issued a certificate of participation. To participate in this journal CME activity: (1) review the learning objectives and author disclosures; (2) study the education content; (3) take the post-test and/or complete the evaluation at www.medscape.org/journal/eid; (4) view/print certificate.

**Release date: February 23, 2011; Expiration date: February 23, 2012**

## Learning Objectives

Upon completion of this activity, participants will be able to:

- Describe the risk for health care workers (HCWs) of developing incident latent tuberculosis infection (LTBI)
- Describe the risk for HCWs of developing tuberculosis (TB) disease
- Describe TB infection control measures that may be effective in healthcare settings

## MEDSCAPE CME Editor

**Karen L. Foster, MA, **Technical Writer-Editor, *Emerging Infectious Diseases. Karen L. Foster, MA, has disclosed no relevant financial relationships*.

## MEDSCAPE CME AUTHOR

**Laurie Barclay, MD, **freelance writer and reviewer, Medscape, LLC. *Disclosure: Laurie Barclay, MD, has disclosed no relevant financial relationships.*

## AUTHORS

*Disclosures:* ***Iacopo Baussano, MD, MSc, PhD, SpID; Brian Williams, PhD; Emanuele Pivetta, MD; Massimiliano Bugiani, MD;*** *and* ***Fabio Scano, MD,*** *have disclosed no relevant financial relationships.* ***Paul Nunn, MA, FRCP(UK),*** *has disclosed the following relevant financial relationship: served as a speaker or a member of a speakers bureau for Howard Hughes Foundation.*
