# Supplementary material for: Tuberculosis among Health Care Workers
Source: Emerg Infect Dis. 2011 Mar;17(3):488–94. doi: 10.3201/eid1703.100947 (PMC3298382; doi:10.3201/eid1703.100947)
Supplement: Technical Appendix — Search Terms, Levins Formula, List of Studies included in the Systematic Review and other technical information. [file 10-0947-Techapp_9p.pdf]

Article DOI: 10.3201/eid1703.100947

Tuberculosis among Health Care Workers

Technical Appendix

Search Terms

(“tuberculosis” or “tuberculosis, multidrug-resistant” or “extensively drug-resistant tuberculosis”) and (“occupational exposure” or “health personnel” or “outpatients” or “nursing homes” or “caregivers” or “physicians” or “medical staff, hospital” or “nurses” or “nurses” aides” or “nurse practitioners” or “students, medical” or “students, nursing” or “health and worker” or “health and care”) and (“cross infection” or “disease transmission” or “disease transmission, professional-to-patient” or “disease transmission, patient-to-professional” or “occupational diseases” or “occupational exposure” or “nosocomial tuberculosis” or “occupational tuberculosis” or “hospital exposure” or “occupational hazard”).

Levin’s Formula

To assess the fraction of tuberculosis in the population that was attributable to the exposure to health care settings, we calculated the PAF using Levin’s formula:

$$PAF\% = \frac{Pe \cdot (IRR - 1)}{1 + Pe \cdot (IRR - 1)} \cdot 100$$

where IRR is the TB IRR measured from each study, and *Pe* is the proportion of the population working in health care settings. As

List of Studies included in the Systematic Review

Twenty-five studies reported latent *Mycobacterium tuberculosis* infection (LTBI) cases among health care workers (HCWs) (Technical Appendix Table 1) (*1–25*). Eighteen studies reported tuberculosis cases among HCWs (Technical Appendix Table 2) (*26–43*). Three studies could not be located and were excluded from the analysis (*44–46*).

References

1. Adal KA, Anglim AM, Palumbo CL, Titus MG, Coyner BJ, Farr BM. The use of high-efficiency particulate air-filter respirators to protect hospital workers from tuberculosis. A cost-effectiveness analysis. N Engl J Med. 1994;331:169–73. Medline DOI: 10.1056/NEJM199407213310306

2. Baussano I, Bugiani M, Carosso A, Mairano D, Pia Barocelli A, Tagna M, et al. Risk of tuberculin conversion among healthcare workers and the adoption of preventive measures. Occup Environ Med. 2007;64:161–6. Medline DOI: 10.1136/oem.2006.028068

3. LoBue PA, Catanzaro A. Effectiveness of a nosocomial tuberculosis control program at an urban teaching hospital. Chest. 1998;113:1184–9. Medline DOI: 10.1378/chest.113.5.1184

4. Liss GM, Khan R, Koven E, Simor AE. Tuberculosis infection among staff at a Canadian community hospital. Infect Control Hosp Epidemiol. 1996;17:29–35. Medline DOI: 10.1086/647185

5. Miller AK, Tepper A, Sieber K. Historical risks of tuberculin skin test conversion among non-physician staff at a large urban hospital. Am J Ind Med. 2002;42:228–35. Medline DOI: 10.1002/ajim.10108

6. Behrman AJ, Shofer FS. Tuberculosis exposure and control in an urban emergency department. Ann Emerg Med. 1998;31:370–5. Medline DOI: 10.1016/S0196-0644(98)70349-X

7. Larsen NM, Biddle CL, Sotir MJ, White N, Parrott P, Blumberg HM. Risk of tuberculin skin test conversion among health care workers: occupational versus community exposure and infection. Clin Infect Dis. 2002;35:796–801. Medline DOI: 10.1086/342333

8. Blumberg HM, Sotir M, Erwin M, Bachman R, Shulman JA. Risk of house staff tuberculin skin test conversion in an area with a high incidence of tuberculosis. Clin Infect Dis. 1998;27:826–33. Medline DOI: 10.1086/514963

9. Zahnow K, Matts JP, Hillman D, Finley E, Brown LS Jr, Torres RA, et al. Rates of tuberculosis infection in healthcare workers providing services to HIV-infected populations. Terry Beirn Community Programs for Clinical Research on AIDS. Infect Control Hosp Epidemiol. 1998;19:829–35. Medline DOI: 10.1086/647740

10. Warren DK, Foley KM, Polish LB, Seiler SM, Fraser VJ. Tuberculin skin testing of physicians at a midwestern teaching hospital: a 6-year prospective study. Clin Infect Dis. 2001;32:1331–7. Medline DOI: 10.1086/319993

11. Menzies D, Fanning A, Yuan L, FitzGerald JM. Factors associated with tuberculin conversion in Canadian microbiology and pathology workers. Am J Respir Crit Care Med. 2003;167:599–602. Medline DOI: 10.1164/rccm.200208-873BC

12. Menzies D, Fanning A, Yuan L, FitzGerald JM. Hospital ventilation and risk for tuberculous infection in canadian health care workers. Canadian Collaborative Group in Nosocomial Transmission of TB. Ann Intern Med. 2000;133:779–89. Medline

13. Levy MZ, Medeiros EA, Shang N, Soares MC, Homenko AS, Almeida RM, et al. TST reversion in a BCG-revaccinated population of nursing and medical students, Sao Paulo, Brazil, 1997-2000. Int J Tuberc Lung Dis. 2005;9:771–6. Medline

14. Silva VM, Cunha AJ, Kritski AL. Tuberculin skin test conversion among medical students at a teaching hospital in Rio de Janeiro, Brazil. Infect Control Hosp Epidemiol. 2002;23:591–4. Medline DOI: 10.1086/501976

15. Maciel EL, Viana MC, Zeitoune RC, Ferreira I, Fregona G, Dietze R. Prevalence and incidence of Mycobacterium tuberculosis infection in nursing students in Vitoria, Espirito Santo. Rev Soc Bras Med Trop. 2005;38:469–72. Medline DOI: 10.1590/S0037-86822005000600004

16. Lopes LK, Teles SA, Souza AC, Rabahi MF, Tipple AF. Tuberculosis risk among nursing professionals from Central Brazil. Am J Infect Control. 2008;36:148–51. Medline DOI: 10.1016/j.ajic.2007.01.013

17. Hohmuth BA, Yamanija JC, Dayal AS, Nardell E, Salazar JJ, Smith Fawzi MC. Latent tuberculosis infection: risks to health care students at a hospital in Lima, Peru. Int J Tuberc Lung Dis. 2006;10:1146–51. Medline

18. Pai M, Gokhale K, Joshi R, Dogra S, Kalantri S, Mendiratta DK, et al. Mycobacterium tuberculosis infection in health care workers in rural India: comparison of a whole-blood interferon gamma assay with tuberculin skin testing. JAMA. 2005;293:2746–55. Medline DOI: 10.1001/jama.293.22.2746

19. Corbett EL, Muzangwa J, Chaka K, Dauya E, Cheung YB, Munyati SS, et al. Nursing and community rates of Mycobacterium tuberculosis infection among students in Harare, Zimbabwe. Clin Infect Dis. 2007;44:317–23. Medline DOI: 10.1086/509926

20. Bonifacio N, Saito M, Gilman RH, Leung F, Cordova Chavez N, Chacaltana Huarcaya J, et al. High risk for tuberculosis in hospital physicians, Peru. Emerg Infect Dis. 2002;8:747–8. Medline

21. Yanai H, Limpakarnjanarat K, Uthaivoravit W, Mastro TD, Mori T, Tappero JW. Risk of Mycobacterium tuberculosis infection and disease among health care workers, Chiang Rai, Thailand. Int J Tuberc Lung Dis. 2003;7:36–45. Medline

22. Rullán JV, Herrera D, Cano R, Moreno V, Godoy P, Peiro EF, et al. Nosocomial transmission of multidrug-resistant Mycobacterium tuberculosis in Spain. Emerg Infect Dis. 1996;2:125–9. Medline DOI: 10.3201/eid0202.960208

23. Roth VR, Garrett DO, Laserson KF, Starling CE, Kritski AL, Medeiros EA, et al. A multicenter evaluation of tuberculin skin test positivity and conversion among health care workers in Brazilian hospitals. Int J Tuberc Lung Dis. 2005;9:1335–42. Medline

24. Lee SS, Liu YC, Huang TS, Chen YS, Tsai HC, Wann SR, et al. Comparison of the interferon-gamma release assay and the tuberculin skin test for contact investigation of tuberculosis in BCG-vaccinated health care workers. Scand J Infect Dis. 2008;40:373–80. Medline DOI: 10.1080/00365540701730743

25. Louther J, Rivera P, Feldman J, Villa N, DeHovitz J, Sepkowitz KA. Risk of tuberculin conversion according to occupation among health care workers at a New York City hospital. Am J Respir Crit Care Med. 1997;156:201–5. Medline

26. Krüüner A, Danilovitsh M, Pehme L, Laisaar T, Hoffner SE, Katila ML. Tuberculosis as an occupational hazard for health care workers in Estonia. Int J Tuberc Lung Dis. 2001;5:170–6. Medline

27. Cuhadaroglu C, Erelel M, Tabak L, Kilicaslan Z. Increased risk of tuberculosis in health care workers: a retrospective survey at a teaching hospital in Istanbul, Turkey. BMC Infect Dis. 2002;2:14. Medline DOI: 10.1186/1471-2334-2-14

28. Skodric-Trifunovic V, Markovic-Denic L, Nagorni-Obradovic L, Vlajinac H, Woeltje KF. The risk of occupational tuberculosis in Serbian health care workers. Int J Tuberc Lung Dis. 2009;13:640–4. Medline

29. Jo KW, Woo JH, Hong Y, Choi CM, Oh YM, Lee SD, et al. Incidence of tuberculosis among health care workers at a private university hospital in South Korea. Int J Tuberc Lung Dis. 2008;12:436–40. Medline

30. Fica CA, Ramonda CP, Jemenao PM, Zambrano GA, Cifuentes DM, Febre VN, et al. [Tuberculosis in health care workers from a public health service in Santiago, Chile]. Rev Chilena Infectol. 2009;26:34–8. Medline

31. Dimitrova B, Hutchings A, Atun R, Drobniewski F, Marchenko G, Zakharova S, et al. Increased risk of tuberculosis among health care workers in Samara Oblast, Russia: analysis of notification data. Int J Tuberc Lung Dis. 2005;9:43–8. Medline

32. Gopinath KG, Siddique S, Kirubakaran H, Shanmugam A, Mathai E, Chandy GM. Tuberculosis among healthcare workers in a tertiary-care hospital in South India. J Hosp Infect. 2004;57:339–42. Medline DOI: 10.1016/j.jhin.2004.03.029

33. Naidoo S, Jinabhai CC. TB in health care workers in KwaZulu-Natal, South Africa. Int J Tuberc Lung Dis. 2006;10:676–82. Medline

34. Alonso-Echanove J, Granich RM, Laszlo A, Chu G, Borja N, Blas R, et al. Occupational transmission of Mycobacterium tuberculosis to health care workers in a university hospital in Lima, Peru. Clin Infect Dis. 2001;33:589–96. Medline DOI: 10.1086/321892

35. Jelip J, Mathew GG, Yusin T, Dony JF, Singh N, Ashaari M, et al. Risk factors of tuberculosis among health care workers in Sabah, Malaysia. Tuberculosis (Edinb). 2004;84:19–23. Medline DOI: 10.1016/j.tube.2003.08.015

36. Harries AD, Kamenya A, Namarika D, Msolomba IW, Salaniponi FM, Nyangulu DS, et al. Delays in diagnosis and treatment of smear-positive tuberculosis and the incidence of tuberculosis in hospital nurses in Blantyre, Malawi. Trans R Soc Trop Med Hyg. 1997;91:15–7. Medline DOI: 10.1016/S0035-9203(97)90376-X

37. Rao KG, Aggarwal AN, Behera D. Tuberculosis among physicians in training. Int J Tuberc Lung Dis. 2004;8:1392–4. Medline

38. Harries AD, Hargreaves NJ, Gausi F, Kwanjana JH, Salaniponi FM. Preventing tuberculosis among health workers in Malawi. Bull World Health Organ. 2002;80:526–31. Medline

39. Harries AD, Nyirenda TE, Banerjee A, Boeree MJ, Salaniponi FM. Tuberculosis in health care workers in Malawi. Trans R Soc Trop Med Hyg. 1999;93:32–5. Medline DOI: 10.1016/S0035-9203(99)90170-0

40. Laniado-Laborín R, Cabrales-Vargas N. Tuberculosis in healthcare workers at a general hospital in Mexico. Infect Control Hosp Epidemiol. 2006;27:449–52. Medline DOI: 10.1086/503641

41. Kanyerere HS, Salaniponi FM. Tuberculosis in health care workers in a central hospital in Malawi. Int J Tuberc Lung Dis. 2003;7:489–92. Medline

42. Wilkinson D, Gilks CF. Increasing frequency of tuberculosis among staff in a South African district hospital: impact of the HIV epidemic on the supply side of health care. Trans R Soc Trop Med Hyg. 1998;92:500–2. Medline DOI: 10.1016/S0035-9203(98)90889-6

43. Torres Costa J, Sa R, Cardoso MJ, Silva R, Ferreira J, Ribeiro C, et al. Tuberculosis screening in Portuguese healthcare workers using the tuberculin skin test and the interferon-gamma release assay. Eur Respir J. 2009;34:1423–8. Medline DOI: 10.1183/09031936.00053809

44. Condos R, Schluger N, Lacouture R, Rom W. Tuberculosis infections among housestaff at Bellevue Hospital in an epidemic period. [abstract]. Am Rev Respir Dis. 1993;147(Suppl):A124.

45. Redwood E, Anderson V, Felton C, Findley S, Ford J. Tuberculin conversions in hospital employees in a high tuberculosis prevalence area. Am Rev Respir Dis ;147:Suppl:A119 abstract. 1993.

46. Lainez RM, Consul M, Olona M, Martinez-Ballarin JI, Miravitlles M, Vidal R. [Tuberculous infection in nursing students: prevalence and conversion during a 3-year follow-up]. Med Clin (Barc). 1999;113:685–9. Medline

Technical Appendix 1 Table 1. Studies reporting incidence of latent TB infection among HCWs, by incidence level, January 2005–July 2010*

Study, country, and reference	Study period	Types of hospital workers	No. cases (no. persons at risk)	ARTI, %†
Incidence <50/100,000				
Adal et al., United States (*1*)	1990–1992	All	23 (124,869)	0.21
Baussano et al., Italy (*2*)	1998–2004	All	146 (8,122)	1.80
LoBue et al., United States (*3*)	1993–1995	All	59 (9,905)	0.60
Liss et al., Canada (*4*)	1991–1994	All	18 (809)	2.22
Miller et al., United States (*5*)	1990–1992	All	138 (2,362)	5.84
Behrman et al., United States (*6*)	1994–1995	All	57 (2,564)	2.22
Larsen et al., United States (*7*)	1994–1998	All	69 (5,773)	1.19
Blumberg et al., United States (*8*)	1992–1997	All	52 (2,144)	2.43
Zahnow et al., United States (*9*)	1992–1995	All	22 (766)	2.87
Warren et al., United States (*10*)	1992–1998	All	21 (731)	2.87
Menzies et al., Canada (*11*)	Not reported	Microbiology and pathology	14 (111)	12.61
Louther et al., United States (*12*)	1991–1994	All	65 (898)	7.24
Menzies et al., Canada (*13*)	1995–1996	All	238 (1,289)	18.46
Rullán et al., Spain (*14*)	1991–1995	All	24 (92)	26.08
Lee et al., South Korea (*15*)	2007	All	16 (196)	8.16
Incidence 50–99/100,000				
Levy et al., Brazil (*16*)	1997–2000	All	1 (46)	2.17
Silva et al., Brazil (*17*)	1998–1999	All	16 (414)	3.86
Roth et al., Brazil (*18*)	1998–1999	All	105 (1209)	8.68
Maciel et al., Brazil (*19*)	1997–1999	All	8 (76)	10.53
Lopes et al., Brazil (*20*)	2001–2004	All	7 (61)	11.48
Incidence ≥100/100,000				
Hohmuth et al., Peru (*21*)	2002–03	Health care students	1 (93)	1.08
Pai et al., India (*22*)	2004	All	6 (147)	4.08
Corbett et al., Zimbabwe (*23*)	2004–2005	Student nurses	41 (213)	19.25
Bonifacio et al., Peru (*24*)	2001	All	5 (35)	14.29
Yanai et al., Thailand (*25*)	1995–1996	All	24 (332)	7.24

*Incidence levels from World Health Organization statistics, 2009 (*8*).TB, tuberculosis; HCW, health care worker; ARTI, annual risk for *Mycobacterium tuberculosis* infection.
†Estimates of ARTI ratio between HCWs and general population, pooled estimates of ARTI ratio (by estimated TB incidence), fraction of the population working as HCW.

Technical Appendix 1 Table 2. Studies reporting TB incidence among HCWs, January 2005–July 2010*

Author, country, and reference	Period	Hospital workers/comparison group	No. cases (no. persons at risk)	TB incidence in HCWs, ×100,000	TB incidence in the comparison, ×100,000	TB IRR† (95% CI)	Incidence difference	HCW population, ×1,000	PAF%
Incidence <50/100,000‡									
Cuhadaroglu C et al., Turkey (*27*)	1991–2000	All/national estimates for Turkey	31 (33,590)	92.29	29	3.18 (2.24–4.53)	63.29	4.47	0.97
Skodric-Trifunovic V et al., Serbia (*28*)	1992–2004	All/national estimates for Serbia§	24 (57,279)	41.9	34	1.23 (0.83–1.84)	8	6.34	0.15
Fica AC et al.,Chile (*30*)	2003–2006	All/national estimates for Chile	12 (30,680)	39.1	23	1.71 (0.97–3.01)	16.26	1.69	0.12
Torres Costa J et al., Portugal (*43*)	2005–2008	All/national estimates for Portugal	31 (16,178)	191.62	32	5.99 (4.21–8.51)	159.62	8.17	3.91
Incidence 50–99/100,000‡									
Dimitrova B et al. (2005), Russia (*31*)	1994–2002	All/national estimates for Russia§	474 (583,695)	81.21	95	0.86 (0.78–0.94)	6.21	12.78	−0.19
Kruuner A et al., Estonia (*26*)	1994–1998	All/national estimates for Estonia§	67 (73,650)	90.97	58	1.38 (1.08–1.75)	32.97	1142	0.43
Jo KW et al., South Korea (*29*)	2001–2006	All/national estimates for South Korea	61 (8,433)	723.35	92	7.86 (6.12–10.11)	631.35	3.50	2.35
Incidence ≥100/100000‡									
Gopinath KG et al., India (*32*)	1992–2001	All/national estimates for India§	125 (60,163)	207.77	168	1.24 (1.04–1.47)	39.77	1.89	0.04
Naidoo S and Jinabhai CC, South Africa (*33*)	2004–2005	All/national estimates for South Africa§	583 (49,392)	1180.35	911	1.30 (1.19–1.41)	462.35	4.85	0.14
Alonso-Echanove J et al., Peru (*34*)	1994–1998	All/national estimates for Peru§	36 (9,200)	391.30	230	1.70 (1.23–2.36)	161.30	1.84	013
Jelip J et al., Malaysia (*35*)	1990–2000	All/national estimates for Malaysia§	205 (73,120)	280.36	114	2.46 (2.14–2.82)	126.36	2.51	0.37
Harries AD et al., Malawi (*36*)	1993–1994	All/ national estimates for Malawi§	12 (620)	1935.48	358	5.41 (3.07–9.52)	1,577.48	0.61	0.27
Rao KG et al., India (*37*)	2001	All/national estimates for India§	13 (1,032)	1259.69	168	7.50 (4.35–12.91)	1,091.69	1.89	1.21
Harries AD et al., Malawi (*38*)	1996–1999	All/national estimates for Malawi§	96 (2,979)	3222.56	405	7.96 (6.51–9.72)	2,817.56	0.61	0.42
Harries AD et al. (1999), Malawi (*39*)	1996	All/national estimates for Malawi§	108 (3,042)	3550.30	389	9.13 (7.56–11.02)	3,161.30	0.61	0.49
Laniado-Laborìn R et al., Mexico *(40*)	1999–2003	All/Tijuana, general population	18 (4,095)	439.56	40	10.99 (6.92–17.44)	399.56	3.34	3.23
Wilkinson D and Gilks CF, South Africa (*42*)	1991–1996	All/national estimates for South Africa§	13 (2,328)	558.42	311	1.80 (1.04–3.09)	247.42	4.85	0.38
Kanyerere HS et al., Malawi (*41*)	2001	All/national estimates for Malawi§	33 (571)	5779.33	414	13.96 (9.92–19.64)	5,360.33	0.61	0.78

*TB, tuberculosis; HCW, health care worker; IRR, incidence rate ratio; CI, confidence interval; PAFP%, fraction of TB in the population attributable to TB exposure in health care settings. WHO, World Health Organization.
†Pooled estimates of annual incidence rate ratio (by estimated TB incidence).
‡World Health Organization statistics, 2009 (*8*).

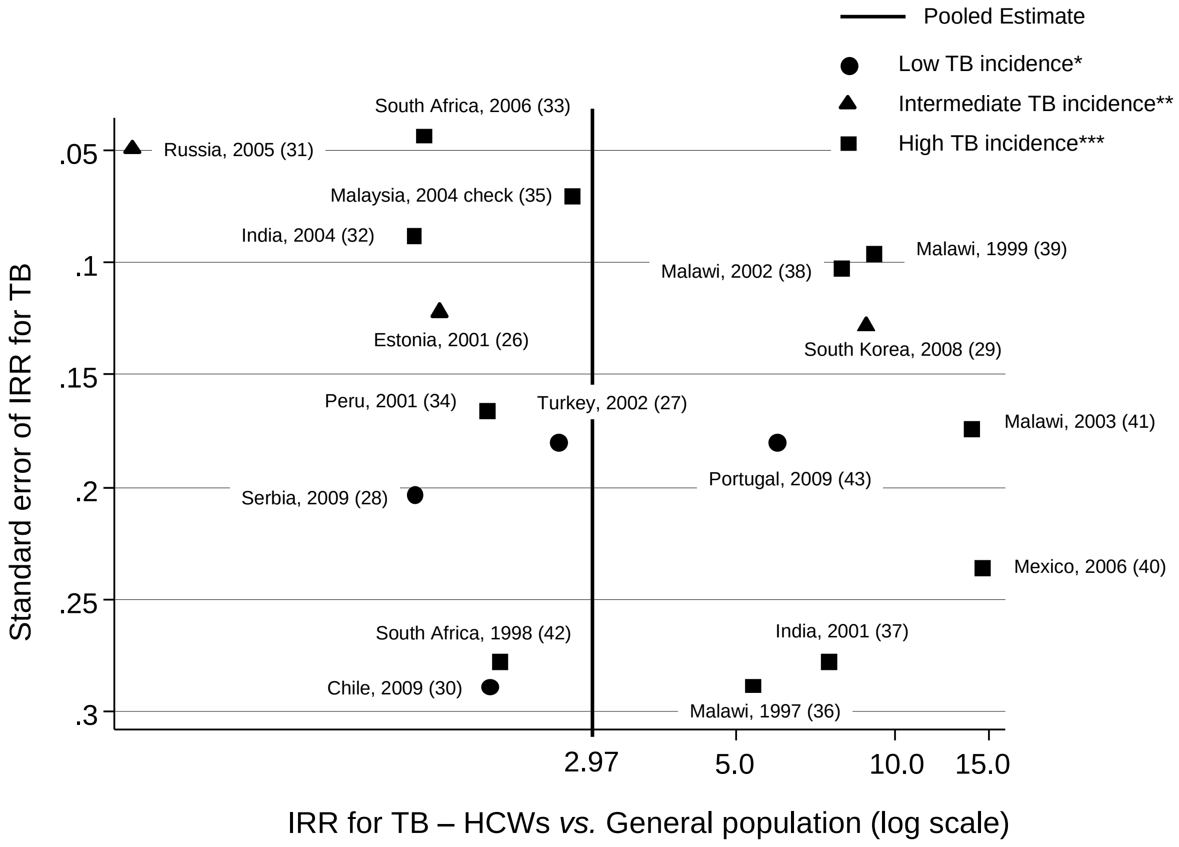

Technical Appendix 1 Figure 1. Funnel Plot for the studies reporting tuberculosis incidence among health care workers. Studies are labeled using the country and year of the study and the reference in parentheses. *<50 cases/100,000 population; **50–99 cases/100,000 population; *** ≥100 cases/100,000 population. TB, tuberculosis; HCW, health care worker; IRR, incidence rate ratio.

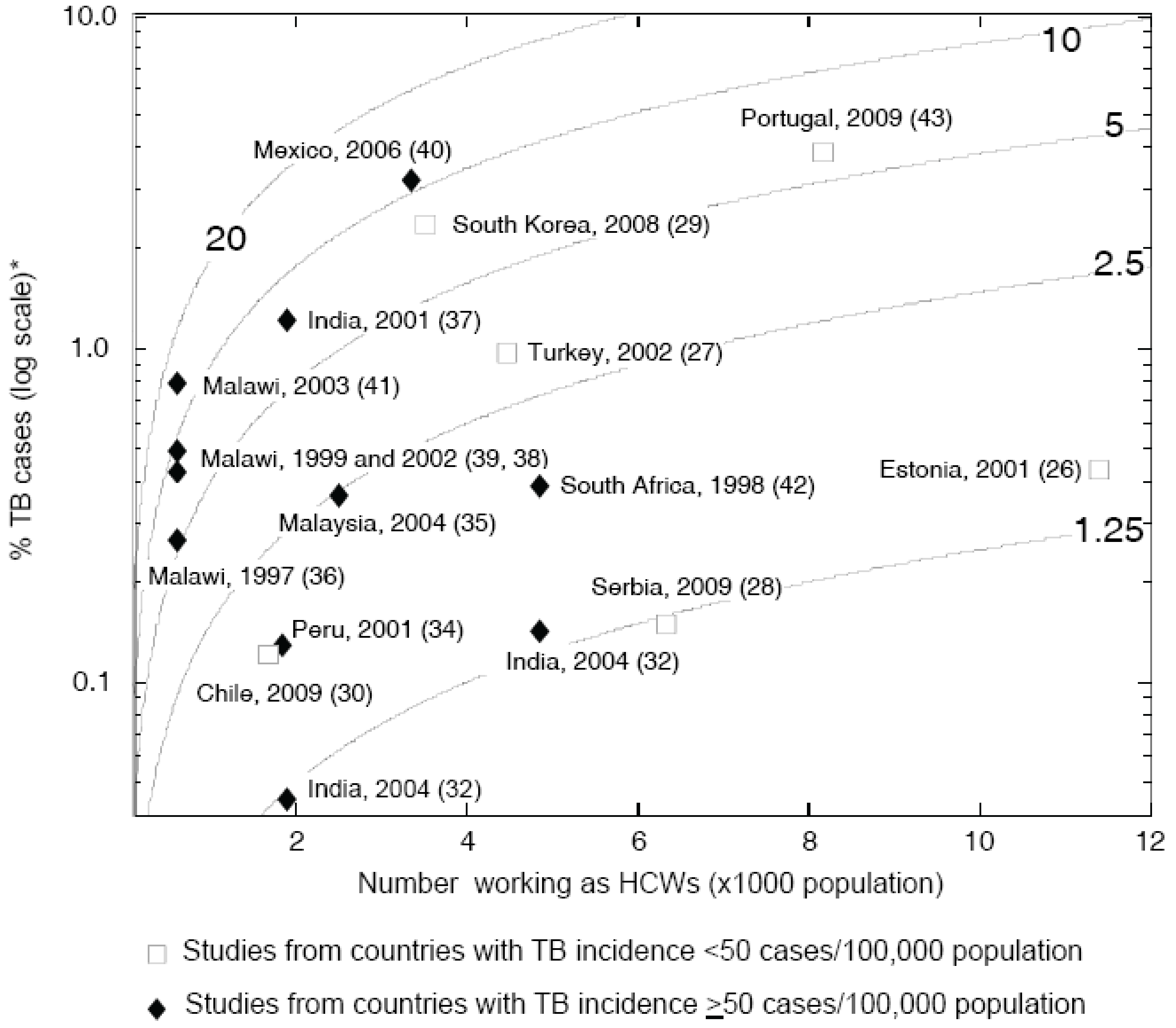

Technical Appendix 1 Figure 2. Contour plot of relationship between the proportion of exposed population, incidence rate ratio (IRR), and population-attributable fraction (PAF) in review of studies about tuberculosis among health care workers. The proportion of the population working in health care settings is reported on the x-azis, y-axis reports the PAF on a log scale calculated by using the Levin formula. The isoclines represent different levels of IRR. The asterisk (*) indicates the fraction (%) of tuberculosis (log scale) in the population attributable to exposure in health care settings. Points are labeled by the country and year of the study, with the reference in parentheses.
