# Supplementary material for: Tuberculosis among Health Care Workers
Source: Emerg Infect Dis. 2011 Mar;17(3):488–94. doi: 10.3201/eid1703.100947 (PMC3298382; doi:10.3201/eid1703.100947)
Supplement: Related texts

# Research article: Tuberculosis among Health Care Workers

**DOI:** 10.3201/eid1703.100947

**Authors:** Iacopo Baussano, Paul Nunn, Brian Williams, Emanuele Pivetta, Massimiliano Bugiani, Fabio Scano

**Affiliations:** Author affiliations: University “A. Avogadro,” Novara, Italy (I. Baussano);; Centro per la Prevenzione Oncologica–Piemonte, Novara (I. Baussano);; Imperial College, London, UK (I. Baussano);; World Health Organization, Geneva, Switzerland (P. Nunn, B. Williams, F. Scano);; University of Turin, Turin, Italy (E. Pivetta);; Centro per la Prevenzione Oncologica–Piemonte, Turin (E. Pivetta);; Regional Reference Centre for Tuberculosis Prevention, Turin (M. Bugiani)

## Abstract

To assess the annual risk for latent tuberculosis infection (LTBI) among health care workers (HCWs), the incidence rate ratio for tuberculosis (TB) among HCWs worldwide, and the population-attributable fraction of TB to exposure of HCWs in their work settings, we reviewed the literature. Stratified pooled estimates for the LTBI rate for countries with low (<50 cases/100,000 population), intermediate (50–100/100,000 population), and high (>100/100,000 population) TB incidence were 3.8% (95% confidence interval [CI] 3.0%–4.6%), 6.9% (95% CI 3.4%–10.3%), and 8.4% (95% CI 2.7%–14.0%), respectively. For TB, estimated incident rate ratios were 2.4 (95% CI 1.2–3.6), 2.4 (95% CI 1.0–3.8), and 3.7 (95% CI 2.9–4.5), respectively. Median estimated population-attributable fraction for TB was as high as 0.4%. HCWs are at higher than average risk for TB. Sound TB infection control measures should be implemented in all health care facilities with patients suspected of having infectious TB.

Transmission of tuberculosis (TB) in health care settings to both patients and health care workers (HCWs) has been reported from virtually every country of the world, regardless of local TB incidence. TB transmission occurs through droplet nuclei aerosolized by patients with infectious pulmonary TB and inhaled by other persons. Transmission is most likely to occur from unrecognized or inappropriately treated TB. The risk for transmission varies by setting, occupational group, local prevalence of TB, patient population, and effectiveness of TB infection control measures (*1*).

In resource-rich countries TB began to be recognized as an occupational hazard in the 1950s (*2*); since then, effective infection control measures have been implemented to reduce the risk for nosocomial TB (*3*). Poor implementation of infection control measures, the effects of the HIV epidemic on TB, and the emergence of multidrug-resistant (MDR) strains resulted in the reemergence of hospital-based transmission of TB and MDR TB in the early 1990s (*2*).

Most recently, the emergence of extensively drug-resistant (XDR) TB strains is posing a major public health threat in contexts characterized by a lack of proper infection control measures (*4*). Although the risk for latent TB infection (LTBI) and TB disease has generally been considered to be higher among HCWs than in the general population, some studies from countries with low TB incidence and others from countries with high TB incidence failed to show a higher estimated risk for TB among HCWs than among the corresponding local community.

We had 2 main objectives in conducting our study. First, by reviewing the literature, we assessed the rate of LTBI and relative risk and risk difference for the incidence of TB among HCWs worldwide, compared with the incidence of TB in the corresponding local general population. Second, we assessed the population-attributable fraction (PAF) (percentage) of TB in the general population associated with exposure to HCWs in their working settings.

## Methods

### Literature Search for Incidence among HCWs

An initial search of the literature for systematic reviews or reviews of meta-analyses estimated LTBI and TB incidence among HCWs worldwide allowed us to identify 4 systematic reviews (*1**,**5**–**7*). Most of the studies analyzed by Menzies et al. in 1995 were performed in the United States, United Kingdom, or Canada (*6*). In 2005, Seidler et al. focused on the risk for TB in low-incidence areas (*7*). In 2006, Joshi et al. analyzed low- and middle-income countries (*5*), and in 2007 Menzies et al. expanded their search and analyses, accounting also for high-income countries (*1*). We identified, retrieved, and considered for inclusion in our study all studies reported in the above-mentioned systematic reviews. To integrate and update the search, we searched 3 electronic databases for primary studies (i.e., PubMed, www.ncbi.nlm.nih.gov/pubmed; EMBASE, http://embase.com; and Web of Knowledge, www.isiwebofknowledge.com) to identify studies that assessed the incidence of LTBI and/or TB among HCWs published during January 2005–July 2010. The combination of terms adopted to perform the search is reported in the Technical Appendix. We restricted our search to articles published in English, French, Spanish, and Italian.

### Study Selection

Studies were eligible for inclusion if they reported the incidence and the corresponding confidence intervals (CIs) of LTBI and TB disease among HCWs or if they reported the number of incident LTBI and TB cases identified in the study along with the overall number of HCWs investigated or the person-years of follow-up. LTBI incidence has been defined as tuberculin conversion after a documented negative-baseline tuberculin skin test (TST) as reported in the original study (*3*). We considered only studies adopting the intradermal TST technique. For cases of TB disease, we have included definitive (microbiologically confirmed) and presumptive (based on clinical, imaging, or pathology) diagnoses.

We excluded studies for a variety of reasons. Studies reporting only case series, only outbreak investigations, or only prevalence of LTBI and TB among HCWs were excluded, as were studies reporting investigations only of MDR TB and of only self-reported LTBI or TB. We also excluded studies that adopted interferon-γ release assay because of paucity of data and potential problems in pooling such data with those obtained from studies that adopted the TST technique. Furthermore, we excluded studies started before 1990 to decrease possible heterogeneity between studies associated with different awareness of TB as an occupational hazard for HCWs (*2*) and studies reporting data from studies investigating only HCWs working in outpatient clinics. All duplicate citations were eliminated from the initial database. Two of the authors (I.B., E.P.) screened these citations by reviewing titles and abstracts to identify potentially relevant studies and discussed disagreements until reaching a consensus. The database was then screened again to include only primary articles, and the full text of each citation was obtained and reviewed.

### Data Extraction

Two of the authors (I.B., E.P.) designed a data extraction form. Then, all articles were independently reviewed and data extraction cross-checked.

The following data were collected from each study: country where the study was performed, study period, and health care setting under investigation. We also collected the estimated incidence of LTBI and/or TB and corresponding CIs and/or number of incident LTBI and/or TB cases identified and the overall number of HCWs investigated or the person-years of follow-up. If reported, the incidence of LTBI and/or TB in a comparison group, such as the local general population or administrative workers not exposed to the health care setting under investigation, was also included. We used estimates provided by the World Health Organization (WHO) for the corresponding study period as estimates of TB incidence among the general populations in the host countries (*8*).

### Data Collation and Meta-analysis

For each study, we considered the incidence for LTBI or TB among HCWs as reported in the original study. Adjusted estimates,when available, were preferred to crude estimates. Alternatively, we calculated the annual incidence of LTBI (hereafter referred to as annual risk for LTBI [ARTI]) and TB among HCWs. Then we estimated the incidence rate ratio (IRR), the risk difference of TB among HCWs compared with the corresponding general population, and the corresponding percentage of TB cases among HCWs attributable to exposure in health care settings. We used WHO estimates for the corresponding period (*8*) for TB incidence in the general population. Three studies could not be located and were excluded from the analysis.

We performed a random effects meta-analysis to account for the expected between-study variability for each study, i.e., we drew pooled estimates under the assumption that each study had different characteristics and measured different, though related, underlying yields (*9*). We used STATA version 11.0 software (StataCorp., College Station, TX, USA) for statistical analysis. The studies included in the meta-analysis were weighted by the inverse variance of their effect-size estimate (*10*).

To assess the fraction of TB in the population attributable to the exposure to health care settings, we calculated the PAF using Levin’s formula (Technical Appendix) (*11*). As a proxy for the proportion of the population working in health care settings, we considered the population of doctors, laboratory workers, nurses, and midwives as reported in WHO’s Global Health Atlas (*8*). Community health workers were not included in this population because no study reported TB cases from these categories. We assessed heterogeneity across studies by the conventional χ^2^ test for heterogeneity and by calculating the I^2^ statistic, which accounts for the number of studies included in the meta-analysis and directly measures the variability not explained by information in the analysis (*12*).

To investigate possible sources of heterogeneity, we stratified the analysis accounting for TB incidence estimated in the country where the study had been conducted. In particular, we defined 3 strata: countries with low TB incidence (i.e., <50 cases/100,000 persons), countries with intermediate TB incidence (i.e., 50–99 cases/100,000 persons), and countries with high TB incidence (i.e., >100 cases/100,000 persons). Furthermore, to formally assess the effect of selected variables, such as the country income level as classified by the World Bank (*13*), the density of HCWs as reported by WHO (*8*), and the year the study was published, as possible sources of heterogeneity, we performed meta-regression analyses in which variance results from 2 additive components, 1 representing the variance within units, and the other the variance between units. Finally, we qualitatively assessed publication bias by drawing a funnel plot (Technical Appendix Figure 1).

**Figure 1:** Flow diagram for selection of studies of tuberculosis (TB) in health care workers (HCWs) published during January 2005–July 2010. INF, interferon.

## Results

We identified 846 potentially relevant unique citations from all literature searches and 74 from previous systematic reviews (Figure 1). Forty-three studies were eligible for inclusion (Technical Appendix); these studies accounted for 1,176 cases of LTBI among HCWs and 49,576 person-years of follow-up and for 1,942 TB cases and >1,010,047 person-years of follow-up. Twenty-five and 18 studies reported LTBI and TB incident cases among HCWs, respectively (Technical Appendix Tables 1, 2).

### LTBI among HCWs

A median of 23 (interquartile range [IQR] 14–59) LTBIs occurred among HCWs. These LTBIs accounted for a median of 731 (IQR 111–2,144) person-years in each study.

The median estimated ARTI among HCWs was 2.9% (IQR 1.8%–8.2%) for studies from countries with low TB incidence, 8.7% (IQR 3.9%–10.5%) for studies from countries with intermediate TB incidence (in the present study only Brazil), and 7.2% (IQR 4.1%–14.3%) for studies from countries with high TB incidence. Consistently, the stratified pooled estimates for the ARTI were 3.8% (95% CI 3.0%–4.6%, I^2^ = 98%), 6.9% (95% CI 3.4%–10.3, I^2^ = 78%), and 8.4% (95% CI 2.7%–14.0%, I^2^ = 89%), respectively (Figure 2). Given that most studies in the meta-analysis were from countries with low TB incidence and that the variability of estimates was higher from countries with intermediate and high TB incidence, the overall (pooled) estimates of ARTI was 4.6% (95% CI 4.1%–5.6%, I^2^ = 97%).

**Figure 2:** Forrest plot showing study-specific and pooled estimates of the annual incidence of latent *Mycobacteria tuberculosis* infection (LTBI) among health care workers (HCWs), by tuberculosis incidence groups according to the World Health Organization. Studies are sorted by incidence category: low, <50 cases/100,000 population; intermediate, 50–99 cases/100,000 population; high, >100 cases/100,000 population. CI, confidence interval. A complete list of references is provided in the Technical Appendix.

### TB among HCWs

A median of 34 (IQR 18–108) TB cases occurred among HCWs; each study accounted for a median of 12,689 (IQR 2,979–57,279) person-years. The median estimated annual incidence of TB among HCWs was 67 cases/100,000 persons (IQR 40–142), 91/100,000 persons (IQR 81–723), and 1,180/100,000 persons (IQR 91–3,222) for studies from countries with low, intermediate, and high TB incidence, respectively. The median estimates for TB incidence for the general population adopted as comparisons were 33/100,000 persons (IQR 27–37), 82/100,000 persons (IQR 58–223), and 311/100,000 persons (IQR 168–405), respectively. Median estimated annual TB IRRs were 2.0 (IQR 1.5–4.1), 1.4 (IQR 0.4–8.8), and 5.4 (IQR 1.7–9.1), respectively.

The median differences in TB incidence for countries with low, intermediate, and high TB incidence were 34 cases/100,000 persons (IQR 12–105), 33/100,000 persons (IQR–41 to 641), and 409/100,000 persons (IQR 166–2817), respectively. These findings suggest that 49%, 27%, and 81% of TB cases, respectively, among HCWs were attributable to exposure in health care settings.

For countries with low and intermediate TB incidence, stratified pooled estimates for annual TB IRRs were similar: 2.42 (95% CI 1.20–3.64, I^2^ = 85%) and 2.45 (95% CI 1.05–3.84, I^2^ = 98%), respectively (Figure 3). The TB IRRs for countries with high TB incidence was higher: 3.68 (95% CI 2.89–4.48, I^2^ = 96%%). The overall estimate of annual TB IRR was 2.97 (95% CI 2.43–3.51, I^2^= 98%).

**Figure 3:** Forrest plot showing the study-specific and pooled estimates of incidence rate ratios (RR) for tuberculosis (TB) among health care workers (HCWs) compared with the corresponding general populations, by TB incidence group according to the World Health Organization. Studies are sorted by incidence category: low, <50 cases/100,000 population; intermediate, 50–99 cases/100,000 population; high, >100 cases/100,000 population. CI, confidence interval. A complete list of references is provided in the Technical Appendix.

For countries with low TB incidence, the proportion of HCWs in the population ranged from 1.7 to 8.2 HCWs per 1,000 residents. For countries with intermediate and high TB incidence, the proportion of HCWs in the population ranged from 3.5 to 12.8 HCWs per 1,000 residents and from 0.6 to 4.8 HCWs per 1,000 residents, respectively.

Using the estimated annual TB IRR and the reported population numbers of HCWs for each country of interest, we estimated the PAF. The median estimated PAF for TB was 0.36% (IQR 0.13–2.24) for studies from countries with low TB incidence, 0.43% (IQR –0.82 to 2.66) for studies from countries with intermediate TB incidence, and 0.38% (IQR 0.14–0.78) for studies from countries with high TB incidence.

For each level of IRR, a nonlinear relationship existed between the PAF and the percentage of HCWs in the population (Technical Appendix Figure 2). For example, Harries et al. (*14*) and Rao et al. (*15*) reported similar IRRs (7.9 and 7.6, respectively), but because of the different proportion of HCWs working in health care settings in Malawi and India, respectively, the estimated PAF varied widely (0.42% and 1.21%, respectively). On the other hand, Gopinath et al. (*16*) and Rao et al. (*15*) reported from India; thus, the proportion of population working in health care settings was assumed to be the same (1.9 HCWs/1,000 persons, respectively) but the large difference in IRR reported in the 2 studies produced a substantial shift in the estimated PAF (from 0.04% to 1.2%).

The meta-regression model, which accounts for both the effect of country income levels as classified by the World Bank (*13*) and for the HCWs densities as reported by WHO (*8*), has shown that the between-studies variance was significantly reduced (τ^2^ decreased from 1.2–0.71). In particular, the IRR for TB among HCWs decreased by ≈10% as the HCW density increased by 1/1,000 persons and by about two thirds in middle-income countries, compared with low-income countries. However, the variability not explained by the information included in the analysis decreased to only 96%. Incorporating the effect of time (i.e., year of publication of the paper) in the analysis did not significantly decrease the heterogeneity between the studies.

Finally, visual inspection of the funnel plot suggested a possible publication bias for the studies reporting TB incidence among HCWs from countries with high TB incidence (Technical Appendix). A formal testing procedure that used the Begg test, however, failed to confirm such a bias (17).

## Discussion

The results of our analysis show that the risk for TB among HCWs is consistently higher than the risk among the general population worldwide. This finding confirms that TB is an occupational disease. The introduction of TB transmission control measures, essential to protect HCWs, may decrease TB annual incidence among HCWs by as much as 49%, 27%, and 81% in countries with low, intermediate, and high TB incidence, respectively.

The annual risk for LTBI among HCWs and the TB IRRs, excluding the study by Dimitrova et al. (*18*), increase according to the background TB incidence in the population. The exceptionally low IRR estimated from the study by Dimitrova et al. may be a consequence of our decision to calculate TB IRR by pooling HCWs working in general health services and in TB facilities and considering as a reference TB incidence in the general population of Russia (95/100,000), which was higher than the incidence reported in the Samara Oblast (75/100,000), where the study was conducted. The reported incidence among HCWs working in general health services was 69/100,000, whereas the incidence among staff at the TB services was 741/100,000, which is 10× higher than among the general population.

The high PAF represents the effect of an increase in the HCW population in a specific setting, as may occur in countries undergoing socioeconomic transition, without the concomitant introduction of TB transmission control measures, and the consequential increased effects of health care settings on the total incidence of TB in the population. In contrast, low PAF estimates might represent the effect of the introduction of TB transmission control measures in health care settings, and the consequential reduced effects of health care settings on total incidence of TB in the population.

Our study suggests that the effect of TB in HCWs (estimated by PAF) on global TB incidence does not exceed 5% and is comparable across countries with different background TB incidence, possibly because the proportion of HCWs and the TB IRRs counterbalance each other. Such estimated PAF figures call for urgent improvement of TB control measures in health care settings, particularly in settings that have a high IRR for TB.

The substantial heterogeneity between studies could be due to differences in methodologic quality, study design, sampling variability, and study populations across studies. In particular, some studies focused specifically on high-risk groups among HCWs, such as laboratory workers (*19**–**21*) and nurses (*22*). Furthermore, we could not discriminate between settings where infection transmission control measures were or were not implemented. It was equally difficult to classify settings according to their risk for admitting a person with unsuspected TB or according to the characteristics of the population attending the health care facilities, such as homeless persons, drug abusers, or migrants from countries with high TB prevalence.

The analysis restricted to the studies reporting cases among HCWs, regardless of their specific professional role, did not show any significant variation of the IRR estimates. Whether the HCWs investigated were representative of the national population of HCWs for each reported study was impossible to know; thus, IRR estimates resulting from the comparison of their risk for TB with the risk for the general population could be nonrepresentative of the national estimates. The density of HCWs and the level of income of each country, considered simultaneously in the analysis, accounted for some between-study variance. The IRR for TB decreased with the proportion of the population working in health care settings and in middle-income countries compared with high-income countries. Unfortunately, however, much of the heterogeneity still remained unexplained.

The method adopted to estimate the PAF typically measures the effects on a population of risk factors for noncommunicable diseases (*11*), ignoring the specific component of TB transmission dynamics between local community and health care settings (*23*). Thus, the PAF estimates presented here should be considered a lower limit for the real effects of controlling TB transmission in health care settings, accounting for the remaining 2 main components acting on such an effect: the proportion of the population working in health care settings and the role of transmission control measures. Furthermore, PAF estimates do not account for patient-to-patient transmission of TB in health care facilities. Patient-to-patient transmission could constitute a major pathway of TB transmission, especially in settings of high HIV prevalence. Control of TB transmission in such a group could appreciably reduce the overall incidence of TB in the general population. However, data on TB transmission among patients are scanty and difficult to analyze in the context of this systematic review.

Although the cost-effectiveness assessment of the introduction of TB transmission control measures, encapsulated in the WHO Stop TB Strategy (24), is beyond the scope of this article, such a potential reduction of TB incidence among HCWs would make attractive a range of infection transmission control strategies. Managerial activities, administrative controls, adoption of N95 respirators, and engineering controls all represent potentially effective measures. The implementation of a sound TB infection control package based on the facility assessment, is hampered by constraints in poor resource settings; nevertheless, administrative controls alone have been proven effective in decreasing the risk for TB among HCWs (*25**–**27*).

In conclusion, our findings show that HCWs are at higher than average risk for infection with *Mycobacterium tuberculosis* and of developing TB disease. For this reason, sound TB infection control measures, including early diagnosis and prompt treatment of infectious cases, should be prioritized. Drug resistance emphasizes the urgency for implementing such measures, which would also benefit reduction of patient-to-patient transmission in health care facilities.

## References

[1] Menzies D, Joshi R, Pai M. Risk of tuberculosis infection and disease associated with work in health care settings. Int J Tuberc Lung Dis. 2007;11:593–605.17519089

[2] Sepkowitz KA. Tuberculosis and the health care worker: a historical perspective. Ann Intern Med. 1994;120:71–9.825045910.7326/0003-4819-120-1-199401010-00012

[3] Jensen PA, Lambert LA, Iademarco MF, Ridzon R. Guidelines for preventing the transmission of *Mycobacterium tuberculosis* in health care settings, 2005. MMWR Recomm Rep. 2005;54(RR-17):1–141.16382216

[4] Wright A, Zignol M, Van Deun A, Falzon D, Gerdes SR, Feldman K, Epidemiology of antituberculosis drug resistance 2002–07: an updated analysis of the Global Project on Anti-Tuberculosis Drug Resistance Surveillance. Lancet. 2009;373:1861–73. 10.1016/S0140-6736(09)60331-719375159

[5] Joshi R, Reingold AL, Menzies D, Pai M. Tuberculosis among health care workers in low- and middle-income countries: a systematic review. PLoS Med. 2006;3:e494. 10.1371/journal.pmed.003049417194191PMC1716189

[6] Menzies D, Fanning A, Yuan L, Fitzgerald M. Tuberculosis among health care workers. N Engl J Med. 1995;332:92–8. 10.1056/NEJM1995011233202067990907

[7] Seidler A, Nienhaus A, Diel R. Review of epidemiological studies on the occupational risk of tuberculosis in low-incidence areas. Respiration. 2005;72:431–46. 10.1159/00008626116088290

[8] World Health Organization. Global health atlas [cited 2009 Feb 16]. http://www.who.int/globalatlas/dataQuery/default.asp

[9] Normand SL. Meta-analysis: formulating, evaluating, combining, and reporting. Stat Med. 1999;18:321–59. 10.1002/(SICI)1097-0258(19990215)18:3<321::AID-SIM28>3.0.CO;2-P10070677

[10] Sterne JAC, Bradburn MJ, Egger M. Meta-analysis in Stata. In: Egger M, Davey-Smith G, Altman DG, editors. Systematic reviews in health care: meta-analysis in context. London: BMJ Publishing Group; 2001. p. 347–69.

[11] Levin ML. The occurrence of lung cancer in man. Acta Unio Int Contra Cancrum. 1953;9:531–41.13124110

[12] Higgins JP, Thompson SG, Deeks JJ, Altman DG. Measuring inconsistency in meta-analyses. BMJ. 2003;327:557–60. 10.1136/bmj.327.7414.55712958120PMC192859

[13] World Bank. World Bank country classification. 2008 [cited 2009 Dec 12]. http://web.worldbank.org

[14] Harries AD, Kamenya A, Namarika D, Msolomba IW, Salaniponi FM, Nyangulu DS, Delays in diagnosis and treatment of smear-positive tuberculosis and the incidence of tuberculosis in hospital nurses in Blantyre, Malawi. Trans R Soc Trop Med Hyg. 1997;91:15–7. 10.1016/S0035-9203(97)90376-X9093617

[15] Rao KG, Aggarwal AN, Behera D. Tuberculosis among physicians in training. Int J Tuberc Lung Dis. 2004;8:1392–4.15581213

[16] Gopinath KG, Siddique S, Kirubakaran H, Shanmugam A, Mathai E, Chandy GM. Tuberculosis among healthcare workers in a tertiary-care hospital in South India. J Hosp Infect. 2004;57:339–42. 10.1016/j.jhin.2004.03.02915262396

[17] Sterne JAC, Egger M, Davey-Smith G. Investigating and dealing with publication and other biases. In: Egger M, Davey-Smith G, Altman DG, editors. Systematic reviews in health care: meta-analysis in context. London: BMJ Publishing Group; 2001. p. 189–208.

[18] Dimitrova B, Hutchings A, Atun R, Drobniewski F, Marchenko G, Zakharova S, Increased risk of tuberculosis among health care workers in Samara Oblast, Russia: analysis of notification data. Int J Tuberc Lung Dis. 2005;9:43–8.15675549

[19] Boudreau AY, Baron SL, Steenland NK, Van Gilder TJ, Decker JA, Galson SK, Occupational risk of *Mycobacterium tuberculosis* infection in hospital workers. Am J Ind Med. 1997;32:528–34. 10.1002/(SICI)1097-0274(199711)32:5<528::AID-AJIM14>3.0.CO;2-49327078

[20] Kim SJ, Lee SH, Kim IS, Kim HJ, Kim SK, Rieder HL. Risk of occupational tuberculosis in National Tuberculosis Programme laboratories in Korea. Int J Tuberc Lung Dis. 2007;11:138–42.17263282

[21] Menzies D, Fanning A, Yuan L, FitzGerald JM. Factors associated with tuberculin conversion in Canadian microbiology and pathology workers. Am J Respir Crit Care Med. 2003;167:599–602. 10.1164/rccm.200208-873BC12446271

[22] Babus V. Tuberculosis morbidity risk in medical nurses in specialized institutions for the treatment of lung diseases in Zagreb. Int J Tuberc Lung Dis. 1997;1:254–8.9432373

[23] Angie B, Ann A, Malgosia G, Michael K, Hans K, Michael L, Tuberculosis control in prisons: a manual for programme managers. Geneva: World Health Organization; 2000.

[24] Raviglione MC, Uplekar MW. WHO’s new Stop TB Strategy. Lancet. 2006;367:952–5. 10.1016/S0140-6736(06)68392-X16546550

[25] Scano F. WHO policy on TB infection control in health care facilities, congregate settings and households. Geneva: World Health Organization; 2009.24432438

[26] Fica CA, Cifuentes DM, Ajenjo HM, Jemenao PM, Zambrano OA, Febre VN, Tuberculosis in health care workers [in Spanish]. Rev Chilena Infectol. 2008;25:243–55.18769770

[27] Yanai H, Limpakarnjanarat K, Uthaivoravit W, Mastro TD, Mori T, Tappero JW. Risk of *Mycobacterium tuberculosis* infection and disease among health care workers, Chiang Rai, Thailand. Int J Tuberc Lung Dis. 2003;7:36–45.12701833

# Research article: Tuberculosis among Health Care Workers

**DOI:** 10.3201/eid1703.100947

**Authors:** Iacopo Baussano, Paul Nunn, Brian Williams, Emanuele Pivetta, Massimiliano Bugiani, Fabio Scano

**Affiliations:** Author affiliations: University “A. Avogadro,” Novara, Italy (I. Baussano);; Centro per la Prevenzione Oncologica–Piemonte, Novara (I. Baussano);; Imperial College, London, UK (I. Baussano);; World Health Organization, Geneva, Switzerland (P. Nunn, B. Williams, F. Scano);; University of Turin, Turin, Italy (E. Pivetta);; Centro per la Prevenzione Oncologica–Piemonte, Turin (E. Pivetta);; Regional Reference Centre for Tuberculosis Prevention, Turin (M. Bugiani)

## Earning CME Credit

To obtain credit, you should first read the journal article. After reading the article, you should be able to answer the following, related, multiple-choice questions. To complete the questions and earn continuing medical education (CME) credit, please go to www.medscape.org/journal/eid. Credit cannot be obtained for tests completed on paper, although you may use the worksheet below to keep a record of your answers. You must be a registered user on Medscape.com. If you are not registered on Medscape.com, please click on the New Users: Free Registration link on the left hand side of the website to register. Only one answer is correct for each question. Once you successfully answer all post-test questions you will be able to view and/or print your certificate. For questions regarding the content of this activity, contact the accredited provider, CME@medscape.net. For technical assistance, contact CME@webmd.net. American Medical Association’s Physician’s Recognition Award (AMA PRA) credits are accepted in the US as evidence of participation in CME activities. For further information on this award, please refer to http://www.ama-assn.org/ama/pub/category/2922.html. The AMA has determined that physicians not licensed in the US who participate in this CME activity are eligible for *AMA PRA Category 1 Credits*™. Through agreements that the AMA has made with agencies in some countries, AMA PRA credit is acceptable as evidence of participation in CME activities. If you are not licensed in the US and want to obtain an AMA PRA CME credit, please complete the questions online, print the certificate and present it to your national medical association.

## Article Title: Tuberculosis among Health Care Workers

## CME Questions

**1. Based on the above meta-analysis by Dr. Baussano and colleagues, which of the following statements about the risk for healthcare workers (HCWs) of developing incident latent tuberculosis infection (LTBI) is most likely correct?**A. There is no clear evidence that TB is an occupational diseaseB. Annual risk for LTBI among HCWs is not associated with background TB incidence in the populationC. Stratified pooled estimates for the LTBI rate for the countries with an estimated low(<50 cases/100,000) TB incidence was 1.8%D. Stratified pooled estimates for the LTBI rate for the countries with an estimated high(>100 cases/100,000) TB incidence was 8.4%**2. You are asked by the World Health Organization to consult on expected risk of developing TB disease for HCWs worldwide. Based on the above meta-analysis, which of the following statements would be most likely to appear in the report of your findings?**A. The background TB incidence in the population is not associated with incidence rate ratios (IRRs) for TB diseaseB. Median estimated population attributable fraction (PAF) percentage for TB disease exceeds 2%C. For TB disease, IRR estimate is 2.4 for countries with estimated low or intermediate TB incidence, and 3.7 for countries with estimated high TB incidenceD. The impact of TB in HCWs on global TB burden exceeds 10%**3. As the consultant described in Question 2, which of the following statements about suggested TB infection control measures is most likely to appear in your report, based on the above meta-analysis?**A. Infection transmission control strategies are not likely to be effectiveB. Implementing TB infection control measures is recommended only at select healthcare facilities caring for patients suspected of having infectious TBC. Administrative controls alone are ineffective in reducing the risk for TB among HCWsD. Managerial activities, administrative controls, N95 respirators, and engineering controls are all potentially effective in reducing the risk for TB among HCWs

## Activity Evaluation

**Table:** 

1. The activity supported the learning objectives.				
Strongly Disagree				Strongly Agree
1	2	3	4	5
2. The material was organized clearly for learning to occur.				
Strongly Disagree				Strongly Agree
1	2	3	4	5
3. The content learned from this activity will impact my practice.				
Strongly Disagree				Strongly Agree
1	2	3	4	5
4. The activity was presented objectively and free of commercial bias.				
Strongly Disagree				Strongly Agree
1	2	3	4	5

## Supplementary Material

Technical Appendix Search Terms, Levins Formula, List of Studies included in the Systematic Review and other technical information.
